# Cannabidiol and Neurodevelopmental Disorders in Children

**DOI:** 10.3389/fpsyt.2021.643442

**Published:** 2021-05-21

**Authors:** Keith A. Kwan Cheung, Murray D. Mitchell, Helen S. Heussler

**Affiliations:** ^1^Centre for Children's Health Research, School of Biomedical Sciences, Faculty of Health, Queensland University of Technology, Brisbane, QLD, Australia; ^2^Centre for Clinical Trials in Rare Neurodevelopmental Disorders, Child Development Program, Children's Health Queensland, Brisbane, QLD, Australia; ^3^Centre for Children's Health Research, University of Queensland, Brisbane, QLD, Australia

**Keywords:** anxiety, autism, cannabinoid, cannabidiol, endocannabinoid system, neuroinflammation, neuropsychiatry, paediatrics

## Abstract

Neurodevelopmental and neuropsychiatric disorders (such as autism spectrum disorder) have broad health implications for children, with no definitive cure for the vast majority of them. However, recently medicinal cannabis has been successfully trialled as a treatment to manage many of the patients' symptoms and improve quality of life. The cannabinoid cannabidiol, in particular, has been reported to be safe and well-tolerated with a plethora of anticonvulsant, anxiolytic and anti-inflammatory properties. Lately, the current consensus is that the endocannabinoid system is a crucial factor in neural development and health; research has found evidence that there are a multitude of signalling pathways involving neurotransmitters and the endocannabinoid system by which cannabinoids could potentially exert their therapeutic effects. A better understanding of the cannabinoids' mechanisms of action should lead to improved treatments for neurodevelopmental disorders.

## Neurodevelopmental and Psychiatric Disorders

Neurodevelopmental disorders in children have profound impacts on the functioning of children and families particularly where an additional mental health diagnosis is present. The prevalence of any neurodevelopmental disorder seems to vary depending on the study; however, it seems to be around 15% of children (3–17 years) in the United States of America (USA) based on parental concerns (1). This includes diagnoses such as Attention Deficit Hyperactivity Disorder (ADHD), Autism Spectrum Disorder (ASD), Intellectual Disabilities (ID) and syndromic disabilities. According to Boyle et al., around 4% of affected children had at least 2 diagnoses. Those who have a neurodevelopmental disorder are often two to four times more at risk of developing a mental health problem than a typically developing child (1). Neurodevelopmental disorders can include anxiety and mood disorders, Tourette's syndrome, psychosis, and bipolar disorders. Individuals with neuroatypical presentations may pose particular challenges to assessment and understanding of the psychiatric diagnosis. They may resort to behavioural escalations (such as tantrums and self-injury) as a manifestation of their extreme distress and inability to communicate their distress and, as such, can be very difficult for families and communities to support (2, 3).

The aetiology of neurodevelopmental disorders is multifactorial with polygenic risk as well as the impact of perinatal exposures to biological or environmental factors that may act as epigenetic modifiers of neuronal networks and structures. The biological underpinning of many of these disorders is only, in part, minimally understood and thus therapies are usually based on responses in typically developing individuals, older paediatric populations and adults. Treatment options for comorbid mental health problems are limited on the whole to symptomatic therapies and often evidence is restricted in these populations as to the treatment's effectiveness and the mechanisms involved. For example, stimulants for ADHD and some newer therapies are frequently used where attention and impulsivity issues are present in other, non-ADHD disorders while anxiety medication may be trialled off-label on a child with ID diagnosed with significant anxiety. Interestingly, a common trait of ASD and ASD-related disorders (such as Fragile X syndrome and 22q11.2 deletion syndrome) is anxiety and seizures (with or without epilepsy) (4–7). The use of atypical antipsychotics continues to be one of the only evidence-based treatments in children with autism and escalated behaviour; however, the side effect profile of antipsychotics is very difficult to manage, which relegates them to be used only as a short-term last resort. Clinicians are regularly trialling medication to support children and families in significant distress, leading to most of these medications to be prescribed off-label for neuroatypical children; therefore, new medications with clear relationships to aetiology and biological underpinnings are required to support these individuals as they develop into adulthood.

## Cannabinoids as Potentially Therapeutic for Paediatric Psychiatric Disorders

There has been interest for a long time in the impact of medicinal cannabis on neurological and psychiatric disorders (8). Phytocannabinoids (cannabinoids) have been found to be molecules that could be pharmaceutically beneficial for some ailments (9). However, the prescription of medical cannabis has been very conservative because of its stigma as a substance of abuse in many jurisdictions (10). Thanks to some well-publicised case studies, a recent increase in community acceptance of cannabis's medical benefits (11) has been shifting government policy in favour of cannabis decriminalisation/legalisation in jurisdictions such as Canada, Israel, Uruguay, a majority of USA states, and the Food and Drug Administration (12–15). In Australia, the Therapeutic Goods Administration (TGA) currently allows strict, limited prescription of medical cannabis by registered medical practitioners (16), and in 2019 the Australian Capital Territory legalised the individual possession and cultivation of small amounts of cannabis (17). Consequently, this surge in therapeutic cannabinoid usage is encouraging a rise in cannabis research, as the cannabis farming industry, biotechnology and pharmaceutical corporations compete to develop more medical cannabinoid products and better commercialise their usage.

Among the 126 cannabinoids in the cannabis plant and its many variants (18), only delta-9-tetrahydrocannabinol (Δ9-THC or THC) is strongly psychoactive and its effects on the developing brain have been a concern for many clinicians as it can induce short-term alterations in mood, behaviour, appetite and cognition (19). Pathological and behavioural aberrations have been detected in chronic cannabis users and can vary with individuals as well as over time (20, 21), making the effects of long-term cannabis treatment on individuals difficult to predict with current methodology. The neurodevelopment of children and adolescents can be disrupted by the cannabinoids' wide-ranging effects on the central nervous system (CNS) (22). The uncertainty of THC's long-term safety has directed society's contemporary focus on cannabidiol (CBD) as the most promising therapeutic cannabinoid due to its relative abundance in the plant, lack of psychoactive effects, positive safety profile (23) and purported benefits (24). There are some synergies between THC and CBD [i.e., THC can reinforce CBD's beneficial properties while CBD dampens THC's psychotropic effects (25, 26)], but THC's psychoactive properties and strong neural interactions can be detrimental after long-term frequent exposure, especially in the developing brain. Indeed, significant alterations in brain structure/function have been observed in humans, adult and adolescent rodents (27–31) frequently consuming cannabis compared to cannabis-free controls. But there is no definitive consensus as other experiments have either reported no significant difference in brain morphology (32) or have been contradictory; for example, one study found thinner brain cortices in adolescent/young adult cannabis users (33) while another study reported increased cortical thickness in adolescent cannabis users (34), compared to non-users of cannabis. Such uncertainty about the long-term effects of cannabinoids on the human brain reinforces the need for in-depth investigations of the cannabinoids' positive and negative effects. There is still very little understanding of how the intake of THC, CBD, and/or other cannabinoids may affect developing neurodivergent brains and research is urgently needed as the use of medicinal cannabis becomes legalised in various parts of the world.

The precise mechanisms behind CBD's beneficial effects are currently not well-understood. CBD does not significantly interact with the cannabinoid receptors that THC interacts strongly with, and its actions have been attributed to inhibition of anandamide degradation (35), serotoninergic, anti-inflammatory and/or its antioxidant properties (36–39). Therapeutic administration of CBD has been demonstrated to alleviate a range of neuropsychiatric symptoms in schizophrenia (35, 40, 41), depression (42) and anxiety (24, 43, 44) (Table 1 summarises a selection of experiments/trials). Encouraged by these findings, CBD therapy has recently been clinically tested in case studies of autism. Aran (3) and Barchel et al. (46) reported improvements in behaviour, anxiety, and communication in oral CBD treatment trials with ASD children—about 60–70% of patients responding well to the treatment, with the side-effects of somnolence and appetite loss being reasonably tolerated. Phase 1b-2 trials of CBD therapy in ASD have demonstrated a positive response in irritability scales on the Aberrant Behaviour Checklist-Community (ABC-C) as well as some core features such as hyperactivity, anxiety. Other trials in phase 2 and phase 3 are underway for anxiety/ behavioural outcomes in ASD, 22q11.2 deletion syndrome (22QS), ID and Tourette's syndrome, with the results of phase 3 studies being awaited. In the case of Fragile X syndrome (FXS) treatment, positive results have also been obtained with successful case studies (5) and clinical trials (45) that involved the participation of children; the studies reported clinically significant improvements in emotional and behavioural symptoms of FXS, namely anxiety, social avoidance, and irritability. The CBD in Heussler's study was administered by transdermal application of a CBD gel patented by Zynerba Pharmaceuticals (4). Most side-effects were mild enough for this novel CBD treatment to be deemed tolerable by the FXS patients (45). Unlike ASD and FXS, there have been no reports published on the efficacy of CBD treatment on 22QS patients as of the time of writing. There is an ongoing clinical trial sponsored by Zynerba Pharmaceuticals, where the efficacy of their CBD gel is being tested on 22QS minors. Due to the commonalities shared by ASD, FXS, and 22QS, the rationale is that CBD would exert anxiolytic and behavioural improvements, resembling those observed in CBD therapy of ASD and FXS (4, 45).

**Table 1:** Summarised findings of some referenced experiments/clinical trials in humans, which demonstrate the wide range of neurological disorders that CBD therapy could potentially be effective for.

**References**	**Disorder**	**Experimental/clinical model**	**Drug dose and route**	**Major findings**
Leweke et al. (35)	Schizophrenia	42 adult schizophrenic patients	800 mg/d, oral	Alleviation of psychotic symptoms
Heussler et al. (45)	Fragile X syndrome	20 FXS patients, aged 6–17 years	Daily 50 mg dose, twice daily 50 mg dose or twice daily 125 mg dose, transdermal	Significant reductions in anxiety and behavioural symptoms
Barchel et al. (46)	Autism Spectrum Disorder	53 children diagnosed with ASD	16 mg/kg/d (maximum of 600 mg), oral	Alleviation of some ASD comorbidity symptoms
Solowij et al. (42)	Depression	20 adult frequent cannabis users	200 mg/d, oral	Significant decrease in depressive and psychotic-like symptoms
Shannon et al. (43)	Anxiety and sleep	72 adults presenting with high anxiety or poor sleep	25 mg/d (maximum of 175 mg for 1 patient), oral	Long-term decrease in anxiety scores within the 1st month of treatment
Devinsky et al. (47)	Refractory epilepsy	120 children and young adults with Dravet syndrome and refractory seizures	5–20 mg/kg/d, oral	Reduction in convulsive-seizure frequency, but higher rates of adverse events than placebo

With many cases of epilepsy persistently resistant to the most common treatment options (48), families of affected epileptic individuals have advocated for the use of medical cannabis as an alternative treatment. CBD demonstrably acts on brain regions and neural pathways in animal and human models of epilepsy via anticonvulsant and neuroprotective effects (38, 49–52). Therefore, cannabinoids (particularly CBD) have been trialled for the management of epilepsy. Paediatric clinical trials are underway in many parts of the world to evaluate pharmaceutical CBD and its impact on a number of areas including completed randomised clinical trials in Dravet and Lennox-Gastaut syndromes (refractory epilepsy syndromes). Two trials focused on the treatment of Lennox-Gastaut syndrome while one trial selected patients affected by Dravet syndrome. All trials had participants regularly administered with a patented oral formulation of 98% CBD (Epidiolex® by GW Pharmaceuticals). In these trials, the participants' pre-existing treatment regime (including medications and/or interventions for epilepsy, such as a ketogenic diet and vagus nerve stimulation) remained unchanged throughout. According to these trials' findings (47, 53, 54), CBD-based pharmaceutical formulations show promise as effective supplementary anticonvulsants, especially to treat refractory epilepsy (55, 56).

Cannabinoid researchers are still attempting to determine the precise effects of each cannabinoid on the human body, and their interactions with each other as well as other xenobiotics (25). Challenges in developing the evidence base for clinical prescribing have been related to products of variable quality with minimal understanding of how various cannabinoids work either individually, together (entourage effect) or with other drugs. One of the ways by which the cannabinoids have been demonstrated to exert their effects is by their direct and indirect interactions with a crucial component of the CNS, called the endocannabinoid system (ECS) (57–59). The ECS is intrinsically linked to neuromodulation, and therefore may be critical in alleviating some neuropsychiatric symptoms (44, 60).

## A Brief Introduction to the Endocannabinoid System

The ECS is a major axis of the CNS, primarily responsible for modulating excitatory and inhibitory synaptic activity through the release of endogenous cannabinoids (endocannabinoids) that interacts with cannabinoid (and non-cannabinoid) receptors (61). Critical features of neural development/health and synaptic plasticity are regulated by the ECS (62). The lipid-based endocannabinoids are secreted extracellularly from the post- to the pre-synaptic site where they bind to cannabinoid receptors to initiate retrograde synaptic signalling (i.e., a negative feedback mechanism that regulates pre-synaptic activity) (63). The cannabinoid receptors, belonging to the G-protein coupled receptor (GPR) family, are found throughout the entire human body—the most well-characterised receptors being the Cannabinoid 1, Cannabinoid 2 and GPR55 receptors.

Cannabinoid 1 receptors (CB1Rs) are particularly abundant in the basal ganglial, cerebellar, cortical and hippocampal regions, with the majority of them present on axon terminals and pre-terminal axon segments (61, 64). CB2 receptors (CB2Rs) are normally expressed at much lower levels in the CNS compared to CB1Rs; this receptor is primarily present in microglia, vascular elements, immune cells and some specific neurons (61, 65). However, when the blood-brain barrier (BBB) is disrupted (by insults such as neuroinflammation), CB2R expression levels in the brain increase due to immune cells flooding the CNS (66). The majority of GPR55 receptors are aggregated in the CNS and peripheral nervous system (67, 68), where their activation on neurons can upregulate intracellular calcium release and inhibit potassium release, resulting in increased neuronal excitability (69, 70).

Activation of the cannabinoid receptors by endocannabinoids can trigger downstream signalling, such as ion channel openings, changes in intracellular calcium ion concentrations and regulation of inflammatory pathways (71). The two most well-studied endocannabinoids are N-arachidonoyl-ethanolamine (anandamide or AEA) and 2-arachidonoylglycerol (2-AG). AEA acts as a high-affinity, partial agonist of CB1R, and barely interacts with CB2R while 2-AG is a full agonist at both CBRs with low-to-moderate affinity, with both endocannabinoids being GPR55 agonists (68, 72, 73). At the end of their normal lifecycle, AEA is mostly degraded to arachidonic acid (AA) and ethanolamine by fatty acid amide hydrolase (FAAH) (71), while 2-AG is majorly converted to AA and glycerol by monoacylglycerol lipase (MAGL) (74). Interestingly, AEA is also a full agonist (with a different affinity than for CB1R) of a non-ECS receptor named the Transient Receptor Potential Vanilloid 1 (TRPV1) that regulates extracellular calcium ion secretion and neuronal excitability (75).

Another potential way for the ECS to affect the progression and severity of neuropsychiatric disorders is via the gut-microbiome-brain axis (76, 77). The gut-microbiome–brain axis is constituted of signalling (neural and humoral) pathways that connect the gastrointestinal system (GIS) and its microbiota to the CNS in reciprocal relationships for homeostatic and defensive maintenance of the whole body. ECS receptors, namely CB1R and TRPV1, peroxisome proliferator-activated receptor alpha (PPAR-α) and GPR119 are strongly expressed throughout the gut-brain axis (e.g., intestinal epithelial cells, myenteric and vagal fibres). These receptors affect myenteric neuron activity, vagal and sympathetic nerve function, and the release of gastrointestinal neuropeptides (such as N-acyl amides), which may subsequently have a significant impact on brain neural activity (77).

The gut microbiota produce metabolites that can interact with the ECS (78, 79). The microbes are usually categorised as either deleterious or beneficial (probiotic) to the host organism, depending on their overall effects (80). Commensal microorganism-derived molecules produce neurotransmitters (e.g., serotonin, GABA), as well as ECS-like mediators that are capable of interacting with host ECS receptors; for example, commendamide is analogous to the human signalling molecules N-acyl amides and interacts with ECS GPRs (81). Currently, the exact effects of these ligands are still mostly unknown, but their existence strongly hint at complex layers of interaction between the gut-brain axis and gut microbiota (78).

Components of the ECS can thus strongly modulate behaviour and mood via interactions with underlying neurotransmission and the gut-microbiome-brain axis.

## The Role Of the Endocannabinoid System in Regulating Anxiety

Anxiety is usually manifested in affected individuals as disproportionate startle response, avoidance behaviour, autonomic hyperactivity, increased muscular tension and reduced motion (66). Anxiety is primarily mediated by glutamatergic (excitatory, i.e., increase likelihood of action potentials), serotoninergic and GABAergic (inhibitory, i.e., decrease likelihood of action potentials) pathways. GABA is the main inhibitory neurotransmitter, widespread throughout the cortex and counters the excitatory activity of glutamatergic neurons (82). Excessive anxiety as experienced by patients with anxiety disorders is theorised to be caused by an imbalance between excitatory and inhibitory signalling. Consequently, such an imbalance may lead to cortical hyper-reactivity and behavioural hypersensitivity in ASD. Puts et al. (83) and Sapey-Triomphe et al. (84) found that cortical GABA levels appear to be reduced in children and adults with ASD, respectively, in comparison to those of neurotypical controls (83, 84). However, Kolodny et al. (85) recently reported no differences in cortical concentrations of GABA and glutamate between neurotypical and ASD young adults (85). This discrepancy in findings could be attributed to low participant numbers and small differences in experimental methodologies. The proper functioning of the ECS is also disrupted in FXS. The loss of Fragile X mental retardation protein (which regulates the translation and transport of messenger RNAs in brain neuron dendrites) in FXS seems to impair the glutamate receptor-5 (mGluR5)-dependent 2-AG signalling at excitatory synapses (86). Additionally, administration of AEA in a mice model of FXS (FMR1 knockout mice) reduced social anxiety (87), suggesting a detrimental downregulation of AEA in FXS.

Functional CB1Rs and CB2Rs expressed (88, 89) in GABAergic, dopaminergic, glutamatergic, and serotoninergic neurons (90–93), could be crucial in regulating behavioural and emotional states (88, 89), which are heavily disrupted in psychiatric/mood disorders. CB1Rs, in particular, are highly expressed on GABAergic interneurons (90, 94), on glutamatergic terminals (90, 92) and on dopamine D1 receptor positive neurons (95). Agonism of CB1Rs can inhibit the secretion of GABA and glutamate from presynaptic terminals (96–99), which indicate that endocannabinoid activation of CB1R can influence the type of synaptic signalling. AEA-mediated TRPV1 activation is linked to an anxiogenic response, as opposed to the anxiolytic response elicited by AEA-mediated CB1R activation. This suggests that there might be an imbalance between CB1R and TRPV1 expression that might play a part in instilling excessive anxiety (100). Inhibition of FAAH by selective inhibitor URB597 was reported to activate serotoninergic neurons in the midbrain of stressed rats, by the associated increase in AEA-mediated signalling at CB1R (101). Inhibition of FAAH and MAGL by selective inhibitors produced anxiolytic effects in CB1R-deficient mice, but not in CB2R-deficient mice, suggesting that CB2R could play a role in regulating anxiety (102). Additionally, CB2R might play a role in regulating anxiety as augmented activation of CB2R by accumulation of 2-AG (via inhibition of MAGL) was found to exert anxiolytic effects in a rat model of stress (103).

From the evidence gathered so far, therapeutic modulation of synaptic signalling and plasticity could indeed be feasible by regulation of the ECS. Moreover, a well-regulated ECS is critical in ensuring good neural health and function as distressed neural cells can lead to further neurological issues such as epilepsy (104).

## How the Ecs Could Be Involved in Neuroinflammation and Epilepsy

The ECS is an important signalling axis for inflammatory pathways throughout the body. Many children affected by ASD, FXS, and 22QS suffer from epileptic/non-epileptic seizures that stem from detrimental mutations responsible for their disorders (5, 7, 105–107). 10–30% of people with ASD have comorbid epilepsy and several synaptic plasticity pathways appear to be involved in both disorders (105). As such, affected children are at increased risk of serious seizure-related accidents and have their neurodevelopment further impaired by frequent seizures (108). In recent years, epilepsy has been surmised to be strongly correlated with neuroinflammation (104, 109). Additionally, abnormally high levels of neuroinflammation have been associated with ASD (110); Vargas et al. (110) and Jyonouchi et al. (111) found higher levels of proinflammatory cytokines (e.g., tumour growth factor–β1) in the brain tissue, cerebrospinal fluid and peripheral blood of ASD patients (including children) (110, 111).

Neuroinflammation is the term given to a set of defensive responses to insult and/or injury in the neural environment that is mainly mediated by glial cells. The resident immune cells of the CNS, the microglia, primarily function in protecting the neuronal population; they are called into action by inflammatory stimuli such as foreign bodies, products from injured/inflamed neurons, blood-brain barrier disruptions, and by chemokines/cytokines [e.g., Interleukin-1β (IL-1β), tumour necrosis factor-α (TNF-α)] (112–114). Neuroinflammation is a protective physiological process but can be harmful when it is excessive and unregulated (115). Multiple parts of the ECS are involved in inflammatory pathways. Moreover, microglia express many components of the ECS (such as CB1R, CB2R and GPR55), via which they communicate with neurons via expression of endocannabinoids (116, 117). There is evidence of microglial involvement in ASD from both brain tissue immunohistochemistry and positron-emission tomography (PET)-imaging studies which revealed increased neuroinflammation and population of activated microglia in brains of ASD patients (118, 119) compared to non-ASD individuals. Therefore, artificially modulating microglial endocannabinoid signalling and treating neuroinflammation could potentially alleviate some ASDsymptoms (117).

Agonism of CB1R and CB2R have shown anti-inflammatory effects in human and animal models (120–123). Antagonism/non-expression of GPR55 also resulted in a reduction in neuronal and microglial inflammation (116, 124, 125). However, agonism of GPR55 in animal and human neural stem cells was found to elicit a neuroprotective effect and rescued neurogenesis after inflammatory insult (126). Additionally, activation of microglial GPR55 by the endogenous ligand l-α-lysophosphatidylinositol limited neuronal damage in rats (127). As Hill et al. suggest, the actions of GPR55 probably strongly depend on the cell type and cause of inflammation (126). Cyclooxygenase enzymes (COX) synthesise signalling intermediaries known as prostanoids, often derived from AA. The constitutive isoform of COX, COX-1, found in numerous cell types, regulates physiological responses, while the inducible isoform, COX-2, is induced rapidly in several cell types (including neurons and glial cells) after biochemical stimuli, such as cytokines and pro-inflammatory molecules (128). COX-2 is involved in the conversion of a minor proportion of AEA and 2-AG to prostaglandin ethanolamides (PG-EAs) (74) and prostaglandin glycerol esters (PG-Gs) (129), respectively—both of which can contribute to inflammatory responses (128). Other prostaglandins derived from AA by COX-1 and COX-2, prostaglandin E_2_ (PGE_2_) and prostaglandin F_2α_ (PGF_2α_), have neurotoxic properties (125, 130, 131). Suppression of MAGL activity (which leads to a downregulation in AA synthesis) has shown neuroprotective effects in mice (132). COX-2 levels have been found to be greatly increased in the brains of patients with epilepsy, compared to non-epileptic patients (133) and in animals that experience prolonged seizures (134), suggesting a relationship between epilepsy and neuroinflammation.

The Cytochrome P450 (CYP) family is another group of enzymes that breaks down endocannabinoids. The ubiquitous CYP enzymes are expressed at different levels across the body, with variations across species and amongst individuals. The CYP enzymes are known for their ability to metabolise xenobiotics, with the metabolites sometimes causing side-effects (135). Changes in CYP activity can influence downstream endocannabinoid signalling pathways by virtue of changes in substrate and metabolite concentrations. CYP3A4, expressed in the human brain (136, 137), derives anti-inflammatory epoxyeicosatrienoic acids (EETs) and pro-inflammatory hydroxyeicosatetraenoic acids (HETEs) from AA (138–141). AEA can be broken down by CYP enzymes (namely CYP3A4, CYP2C19, CYP2D6, and CYP2J2) into EET-ethanolamides (EET-EAs) and HETE-ethanolamides (HETE-EAs) (142–144). Just like their precursor molecules, the EET-EAs and HETE-EAs can bind to CB1Rs and CB2Rs, albeit with different affinities, e.g., 5,6-EET-EA binds much more strongly with CB2R than AEA (145) while 20-HETE-EA and 14,15-EET-EA have only a weak affinity for CB1R (146) in murine models. CYP2J2 breaks down 2-AG to create two products, 2-11,12-epoxyeicosatrienoic glycerol (EET-G), and 2-14,15-EET-G (147), which interact strongly with both CBRs (especially CB1R) (148). Many CYP metabolites therefore are potentially endogenous ligands for some of the ECS receptors and could subsequently be involved in inflammation regulation.

The lipoxygenase (LOX) enzyme pathway is another metabolic route for endocannabinoids and other related fatty acids (149). The LOX pathway starts with the change of AA into leukotriene A4 by the 5-LOX enzyme (expressed on cell types such as neurons). Leukotriene A4 (LTA4) is rapidly catalysed into LTB4 and cysteinyl leukotrienes (i.e., Cys-LTs, which comprises LTC4, LTD4 and LTE4) (149, 150). LTD4 has been linked to blood-brain barrier dysfunction (151), a contributing factor of neuroinflammation (152), as evidenced by exposure of microglial Cys-LT1 and Cys-LT2 receptors to LTD4 resulting in microglial secretion of pro-inflammatory IL-1β in mice (153). In brief, the ongoing research on eicosanoids (collective term for the endocannabinoids and the many metabolites of the ECS) indicates that the ECS is thoroughly implicated in regulation of neuronal activity and neuroinflammation. But until the signalling pathways involved are thoroughly investigated, particularly in the human brain, how neuroinflammation is exactly linked to ECS dysfunction and psychiatric impairments remains to be elucidated. Interestingly, inflammation in the GIS could substantially affect the gut-microbiome-brain axis and subsequent neuronal activity as ASD individuals have been reported to suffer from gastrointestinal issues (such as diarrhoea and constipation) (154–157) and dysbiotic microbiota compared to neurotypical individuals (158). Perturbations in gut microbial diversity has been found to influence neuroinflammation (159) as some gut microbes can secrete pro-inflammatory metabolites and cytokines (160) that cross the blood-brain barrier. CB1R, TRPV1 and PPAR-α can modulate the permeability of the gut-vascular barrier that prevents the entry of intestinal bacteria into the bloodstream; if the GVB's selective permeability is compromised, the bacteria themselves can enter the bloodstream and cross the BBB, causing an inflammatory response (161).

In summary, there is little doubt that the ECS is likely to be central in the aetiology and occurrence of neuropathology, whereby the modulation of the ECS at multiple points by extraneous agents such as cannabinoids could achieve beneficent outcomes.

## Cannabinoids Interact With the Ecs and Neurotransmission

**Figure 1:**
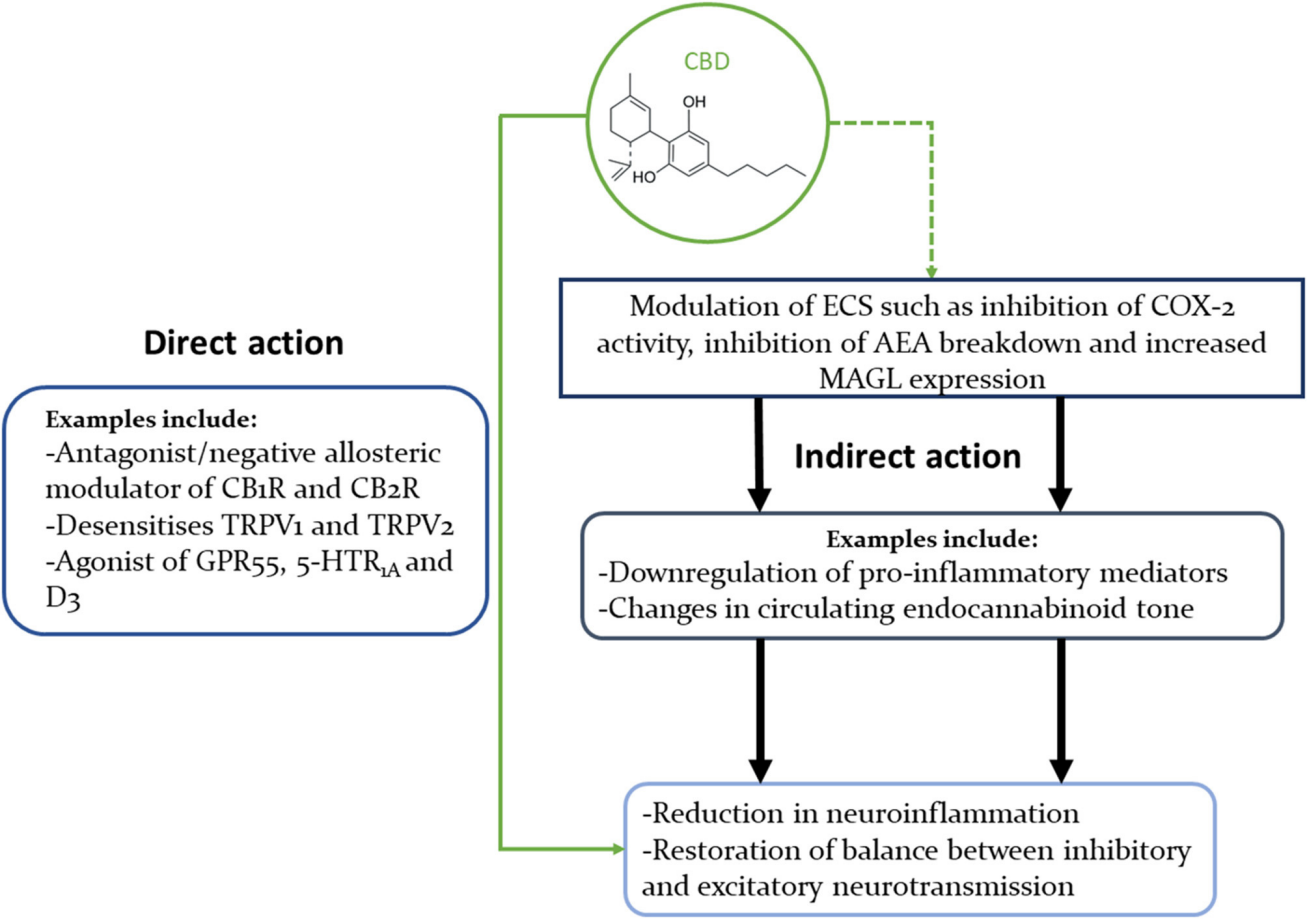
Concise illustration of CBD's interactions with multiple signalling pathways that could explain its beneficial effects in neuropsychiatric disorders. This diagram highlights the fact that CBD can modulate the ECS in multiple ways, as well as interact directly with many neural receptors (only some of which are shown in this diagram).

The cannabinoids' interactions with multiple receptors and enzymes can be safely assumed to hold the key to their wide-ranging therapeutical benefits, but can also obscure the exact mechanisms of their effects. THC is a partial agonist of CB1Rs and CB2Rs, and an agonist of GPR55. On the other hand, CBD's antagonistic/negative allosteric modulating actions on the CB1 and CB2 receptors (57, 162–164) might help explain how CBD can dampen THC's psychoactivity (165) (Figure 1).

While CBD might not interact strongly with CB1R and CB2R when administered at therapeutical levels (166), it has been reported to regulate calcium ion homeostasis in neurons (167) and increase inhibitory neurotransmission via interactions with GPR55 (164). CBD therapy has been correlated with an increase in AEA blood levels and a reduction in the psychotic symptoms of treated schizophrenic patients vs. placebo-control patients (35); the mechanism behind CBD's beneficial effect in this instance could be due to an increase in AEA levels found to be lower in the cerebrospinal fluid of epileptic patients (168) and in the blood of ASD children (169, 170). Of note, the mechanism by which CBD increases AEA levels seems to differ between species; Elmes et al. reported that, in humans, this effect may be due to CBD binding preferentially to the fatty acid binding proteins on which AEA depends to be transported into cells for FAAH catalysis rather than the CBD-induced FAAH inhibition observed in rodents (171). This interaction between CBD and AEA metabolism in humans vs. rodents (171, 172), highlights that the differences in xenobiotics metabolism between species can limit the utility of animal models in cannabinoid research.

In animal models of ASD, an increase in AEA concentration has been correlated with improvements in social interactions. AEA can interact with oxytocin, a neuropeptide that promotes parental and social bonding. Indeed, recent evidence has demonstrated that oxytocin stimulates AEA release in the nucleus accumbens, a key region for the reinforcing properties of natural rewards, with AEA-mediated signalling a requirement for the pro-social effects of this neuropeptide (173). A model of defective oxytocin-driven AEA signalling in ASD could therefore explain how CBD intake ameliorates social interactions in ASD patients (3, 46). Upregulated *Magl* gene (gene that encodes for the MAGL enzyme) expression has been observed in rat hypothalami treated with 10 mg/kg THC (174), supporting the hypothesis that cannabinoids can modulate cerebral endocannabinoid tone. Cannabinoids, like CBD, have been found to inhibit COX-2 activity and hence reduce the production of pro-inflammatory prostaglandins, which could be an additional pathway by which cannabinoids increase the levels of the endocannabinoids, triggering an indirect anti-inflammatory and anti-epileptic activity (175, 176). CBD's inhibition of cerebral CYP isoenzymes could, in turn, modulate the levels of EETs, EET-EAs and HETE-EAs. Therefore, even though CBD may not have a high affinity for CB1R, CB2R, and GPR55, the activation of these endocannabinoid receptors may be indirectly affected by CBD's upregulation/downregulation of endocannabinoids and eicosanoids (136); for example, Bornheim et al. found that CBD inhibited the CYP-driven formation of some AEA metabolites in mice (177) while Arnold et al. reported that THC and CBD inhibited the production of EET-EAs by cardiac CYP2J2 (178). Additionally, the activity and metabolite synthesis of 5-LOX was reduced in human tumour cells treated with CBD (179). Targeted inhibition of Cys-LT synthesis significantly attenuated seizures in treated mice (compared to untreated mice) (180, 181) and in epileptic patients (182), so CBD's inhibition of 5-LOX could have an anti-inflammatory effect.

Intriguingly, CBD has been shown to desensitise non-cannabinoid TRPV1s (75) and related TRPV2s, hence blocking the release of calcium ions outside cells and dampening hyperexcitability (contributor to aberrant neuronal activity) in neurons, suggesting another potential regulatory mechanism (172, 183). CBD has been reported to enhance microglial phagocytosis in rodent microglia partially via the activation of TRPV1 and probably TRPV2 receptor channel of the microglial cells (112); however, Hassan et al. cautioned that increasing microglial phagocytosis might not be a positive strategy for combating neuroinflammation, but their results might not be applicable to human physiology.

As we highlighted beforehand, the cannabinoids may indeed exert their effects differently between species. Another case of CBD's promiscuous interactions is its agonistic actions on the serotonin (5-hydroxytryptamine-1A) receptors (5-HTR_1A_), which are deeply involved in activating anxiolytic responses and in neuronal electrochemical activity (36, 184, 185). In healthy and ASD human adults, CBD suppressed the activity of excitatory glutamatergic neurons in the prefrontal cortex via activation of 5-HTR_1A_ (186), which could contribute to restoring the balance between inhibitory and excitatory neurotransmission. Additionally, CBD inhibits the equilibrative nucleoside transporter (ENT1) responsible for the synaptic uptake of adenosine, thereby increasing levels of extracellular adenosine. Consequently, an upregulation in extracellular adenosine can cascade into a decrease in neuronal hyperexcitability (187–189). CBD has anti-oxidative and anti-inflammatory properties that could counter neuroinflammation; modulation of TRPV1, CB2R, and GPR55 receptors can lead to downregulation of enzymes involved in the production of pro-inflammatory PGs, reactive oxygen species, and cytokines (190, 191). Another potential avenue for CBD's anti-inflammatory action could be its inhibition of voltage-dependent anion selective channel protein 1 (VDAC1) conductance, leading to a decrease in neuroinflammation (192). CBD was also found to enhance the inhibitory γ-Aminobutyric acid (GABA)'s activation of its associated GABA_A_ receptors which regulate inhibitory neurotransmission (193) and are targeted by drugs such as clobazam; indeed, co-administration of CBD with clobazam significantly increased the inhibitory effects of GABA compared to either compound alone (194). Additionally, CBD's amplifying effects on GABA receptors could compensate for the reduced GABAergic transmission observed in FXS (195).

Lower levels of AEA (35) and higher expression/reduced methylation of *CNR1* (the gene coding for CB1R) (196, 197) in schizophrenic patients strongly suggest a pathological link with ECS dysfunction; CBD might compensate for this dysfunction by indirectly modulating endocannabinoid levels. Additionally, CBD is a partial agonist to dopamine D3 receptor, whose expression was demonstrated to be altered in the methylazoxymethanol acetate (MAM) murine neurodevelopmental model (198). Gestational MAM treatment of pregnant dams is a validated model that produces murine offspring with adult phenotype typical of schizophrenia, such as cognitive deficits, dopaminergic dysfunction, physical and behavioural abnormalities (196, 198, 199). Another murine model that mimics the development of the human schizophrenia phenotype is perinatal THC exposure of neonates as it results in similar neurodevelopmental impairments; the cognitive and social deficits were then demonstrated to be reversed by peripubertal CBD treatment (197). These experimental results reinforce the notion that early childhood treatment with CBD might be sufficient to minimise the impact of neurodevelopmental disorders into adulthood.

CBD's interactions with the GIS ECS might depend on the mode of administration; oral intake of CBD is subject to first-pass metabolism, which can result in most of the CBD being transformed by liver enzymes into its metabolites prior to reaching the gut (200). Conversely, more direct passage of CBD in circulating blood via dermal application or inhalation would hypothetically reduce CBD's availability to the GIS. Research on CBD's effects on the gut microbiome and gut ECS are few and limited to animal model studies (generally germ-free mice) (201), but CBD's anti-inflammatory properties could be potentially involved in counteracting gut cell inflammation, gut-vascular barrier leakage and subsequent neuroinflammation by dysbiotic gut microbes (202, 203).

## Conclusion

Our review has hopefully shown that there is a strong body of evidence that early cannabinoid treatment may offer significant potential to safely alleviate many of the common symptoms affecting children with neurodevelopmental disorders. Continued research and evidence in establishing definite relationships between cannabinoid intake and alterations of the ECS are needed to determine clear risk-benefit profiles and to screen for potential individuals in whom benefit could be predicted. CBD is currently the most promising therapeutic cannabinoid for children due to its safety profile and broad-spectrum action. A fuller understanding of CBD's metabolism in the human body (especially how it might interact with the GIS and microbiota) and mechanisms of action could result in greater optimisation of cannabinoid delivery and better development of synthetic cannabinoid analogues.

## Author Contributions

KKC and HH contributed to the conceptualisation and writing of the article. MM the primary supervisor of KKC's research project, also reviewed, and contributed to the manuscript. All authors contributed to the article and approved the submitted version.

## Conflict of Interest

The Centre for Clinical Trials in Rare Neurodevelopmental Disorders of which HH is a Co-Director has conducted sponsored trials for Zynerba Pharmaceuticals, GW Pharmaceuticals, Axial Biotherapeutics, Ovid Therapeutics and Anavex Pharmaceuticals. The remaining authors declare that the research was conducted in the absence of any commercial or financial relationships that could be construed as a potential conflict of interest.

## Abbreviations

22QS: 22q11. 2 deletion syndrome
2-AG: 2-arachidonoylglycerol
5-HTR: 5-hydroxytryptamine receptor
AA: Arachidonic acid
ADHD: Attention Deficit Hyperactivity Disorder
AEA: N-arachidonoyl-ethanolamine or anandamide
AEDs: Anti-epileptic drugs
ASD: Autism Spectrum Disorder
BBB: Blood-brain barrier
CBD: Cannabidiol
CB1R: Cannabinoid receptor 1
CB2R: Cannabinoid receptor 2
Cys-LT: Cysteinyl leukotriene
CNS: Central nervous system
COX: Cyclooxygenase
CYP: Cytochrome P450
EA: Ethanolamide
ECS: Endocannabinoid system
EET: Epoxyeicosatrienoic acid
ENT1: Equilibrative nucleoside transporter
FAAH: Fatty acid amide hydrolase
FDA: Food and Drug Administration
FXS: Fragile X syndrome
GABA: γ-Aminobutyric acid
GPR: G-protein coupled receptor
HETE: Hydroxyeicosatetraenoic acid
HPETE: 5-hydroperoxyeicosatetraenoic acid
ID: Intellectual disabilities
IL-1β: Interleukin-1β
LOX: Lipoxygenase
LT: Leukotriene
MAGL: Monoacylglycerol lipase
MAM: Methylazoxymethanol acetate
PG: Prostaglandin
TGA: Therapeutic Goods Administration
TNF-α: Tumour necrosis factor-α
Δ9-THC or THC: Delta-9 tetrahydrocannabinol
TRPV: Transient Receptor Potential Vanilloid
VDAC1: Voltage-dependent anion selective channel protein 1.

## References

[1] Boyle CA Boulet S Schieve LA Cohen RA Blumberg SJ Yeargin-Allsopp M. Trends in the prevalence of developmental disabilities in US children, 1997-2008. Pediatrics. (2011) 127:1034–42. 10.1542/peds.2010-2989 21606152

[2] Maskey M Warnell F Parr JR Le Couteur A McConachie H. Emotional and behavioural problems in children with autism spectrum disorder. J Autism Dev Disord. (2013) 43:851–9. 10.1007/s10803-012-1622-9 22895777

[3] Aran A Cassuto H Lubotzky A Wattad N Hazan E. Brief report: cannabidiol-rich cannabis in children with autism spectrum disorder and severe behavioral problems—a retrospective feasibility study. J Autism Dev Disord. (2019) 49:1284–8. 10.1007/s10803-018-3808-2 30382443

[4] Sebree T Gutterman D. Treatment of 22q11.2 Deletion Syndrome With Cannabidiol (2019).

[5] Tartaglia N Bonn-Miller M Hagerman R. Treatment of fragile X syndrome with cannabidiol: a case series study and brief review of the literature. Cannabis Cannabinoid Res. (2019) 4:3–9. 10.1089/can.2018.0053 30944868 PMC6446166

[6] Zinkstok JR Boot E Bassett AS Hiroi N Butcher NJ Vingerhoets C. Neurobiological perspective of 22q11.2 deletion syndrome. Lancet Psychiatry. (2019) 6:951–60. 10.1016/S2215-0366(19)30076-8 31395526 PMC7008533

[7] Satterstrom FK Kosmicki JA Wang J Breen MS De Rubeis S An JY. Large-scale exome sequencing study implicates both developmental and functional changes in the neurobiology of autism. Cell. (2020) 180:568–84.e523. 10.1016/j.cell.2019.12.036 31981491 PMC7250485

[8] Russo EB Jiang HE Li X Sutton A Carboni A del Bianco F. Phytochemical and genetic analyses of ancient cannabis from Central Asia. J Exp Bot. (2008) 59:4171–82. 10.1093/jxb/ern260 19036842 PMC2639026

[9] National Academies of Sciences E Medicine. The Health Effects of Cannabis and Cannabinoids: The Current State of Evidence and Recommendations for Research. Washington, DC: The National Academies Press (2017). 28182367

[10] Gloss D Vickrey B. Cannabinoids for epilepsy. Cochrane Database Syst Rev. (2014) 2014:CD009270. 10.1002/14651858.CD009270.pub3 PMC7120304 24595491

[11] Maa E Figi P. The case for medical marijuana in epilepsy. Epilepsia. (2014) 55:783–6. 10.1111/epi.12610 24854149

[12] Hall W Stjepanović D Caulkins J Lynskey M Leung J Campbell G. Public health implications of legalising the production and sale of cannabis for medicinal and recreational use. Lancet. (2019) 394:1580–90. 10.1016/S0140-6736(19)31789-1 31657733

[13] Hill KP George TP. Cannabis legalisation in Canada: a crucial trial balloon. Lancet Psychiatry. (2019) 6:5–6. 10.1016/S2215-0366(18)30460-7 30579495

[14] Destrée L Amiet D Carter A Lee R Lorenzetti V Segrave R. Exploring the association of legalisation status of cannabis with problematic cannabis use and impulsivity in the USA. Drugs Context. (2018) 7:212541. 10.7573/dic.212541 30263037 PMC6152614

[15] Morales P Reggio PH. CBD: a new hope? ACS Med Chem Lett. (2019) 10:694–5. 10.1021/acsmedchemlett.9b00127 31097982 PMC6511966

[16] Martin JH Bonomo YA. Medicinal cannabis in Australia: the missing links. Med J Aust. (2016) 204:371–3. 10.5694/mja16.00234 27256646

[17] Lowrey T. ACT Legalises Personal Cannabis Use, Becoming First Australian Jurisdiction To Do So. Australian Broadcasting Coroporation. (2019). Available online at: https://www.abc.net.au/news/2019-09-25/act-first-jurisdiction-to-legal-personal-cannabis-use/11530104 (accessed September 26, 2019).

[18] Morales P Hurst DP Reggio PH. Molecular targets of the phytocannabinoids: a complex picture. Prog Chem Organic Nat Prod. (2017) 103:103–31. 10.1007/978-3-319-45541-9_4 PMC5345356 28120232

[19] Adams IB Martin BR. Cannabis: pharmacology and toxicology in animals and humans. Addiction. (1996) 91:1585–614. 10.1111/j.1360-0443.1996.tb02264.x 8972919

[20] Huestis MA. Human cannabinoid pharmacokinetics. Chem Biodivers. (2007) 4:1770–804. 10.1002/cbdv.200790152 17712819 PMC2689518

[21] Andréasson S Engström A Allebeck P Rydberg U. Cannabis and schizophrenia a longitudinal study of Swedish conscripts. Lancet. (1987) 330:1483–6. 10.1016/S0140-6736(87)92620-1 2892048

[22] Dow-Edwards D Silva L. Endocannabinoids in brain plasticity: cortical maturation, HPA axis function and behavior. Brain Res. (2017) 1654:157–64. 10.1016/j.brainres.2016.08.037 27569586

[23] Friedman D French JA Maccarrone M. Safety, efficacy, and mechanisms of action of cannabinoids in neurological disorders. Lancet Neurol. (2019) 18:504–12. 10.1016/S1474-4422(19)30032-8 30910443

[24] Micale V Di Marzo V Sulcova A Wotjak CT Drago F. Endocannabinoid system and mood disorders: Priming a target for new therapies. Pharmacol Ther. (2013) 138:18–37. 10.1016/j.pharmthera.2012.12.002 23261685

[25] McPartland JM Duncan M Di Marzo V Pertwee RG. Are cannabidiol and Δ(9) -tetrahydrocannabivarin negative modulators of the endocannabinoid system? A systematic review. Br J Pharmacol. (2015) 172:737–53. 10.1111/bph.12944 25257544 PMC4301686

[26] Niesink RJM van Laar M. Does cannabidiol protect against adverse psychological effects of THC? Front Psychiatry. (2013) 4:130. 10.3389/fpsyt.2013.00130 24137134 PMC3797438

[27] Dalton VS Zavitsanou K. Cannabinoid effects on CB1 receptor density in the adolescent brain: an autoradiographic study using the synthetic cannabinoid HU210. Synapse. (2010) 64:845–54. 10.1002/syn.20801 20842718

[28] Rubino T Prini P Piscitelli F Zamberletti E Trusel M Melis M. Adolescent exposure to THC in female rats disrupts developmental changes in the prefrontal cortex. Neurobiol Dis. (2015) 73:60–9. 10.1016/j.nbd.2014.09.015 25281318

[29] Abush H Akirav I. Short- and long-term cognitive effects of chronic cannabinoids administration in late-adolescence rats. PLoS ONE. (2012) 7:e31731. 10.1371/journal.pone.0031731 22348124 PMC3278466

[30] Boulos PK Dalwani MS Tanabe J Mikulich-Gilbertson SK Banich MT Crowley TJ. Brain cortical thickness differences in adolescent females with substance use disorders. PLoS ONE. (2016) 11:e0152983. 10.1371/journal.pone.0152983 27049765 PMC4822952

[31] Mata I Perez-Iglesias R Roiz-Santiañez R Tordesillas-Gutierrez D Pazos A Gutierrez A. Gyrification brain abnormalities associated with adolescence and early-adulthood cannabis use. Brain Res. (2010) 1317:297–304. 10.1016/j.brainres.2009.12.069 20045399

[32] Chye Y Suo C Lorenzetti V Batalla A Cousijn J Goudriaan AE. Cortical surface morphology in long-term cannabis users: A multi-site MRI study. Eur Neuropsychopharmacol. (2019) 29:257–65. 10.1016/j.euroneuro.2018.11.1110 30558823

[33] Mashhoon Y Sava S Sneider JT Nickerson LD Silveri MM. Cortical thinness and volume differences associated with marijuana abuse in emerging adults. Drug Alcohol Depend. (2015) 155:275–83. 10.1016/j.drugalcdep.2015.06.016 26249265 PMC4581973

[34] Jacobus J Squeglia LM Meruelo AD Castro N Brumback T Giedd JN. Cortical thickness in adolescent marijuana and alcohol users: a three-year prospective study from adolescence to young adulthood. Dev Cogni Neurosci. (2015) 16:101–9. 10.1016/j.dcn.2015.04.006 PMC4624050 25953106

[35] Leweke FM Piomelli D Pahlisch F Muhl D Gerth CW Hoyer C. Cannabidiol enhances anandamide signaling and alleviates psychotic symptoms of schizophrenia. Transl Psychiatry. (2012) 2:e94. 10.1038/tp.2012.15 22832859 PMC3316151

[36] Russo EB Burnett A Hall B Parker KK. Agonistic properties of cannabidiol at 5-HT1a receptors. Neurochem Res. (2005) 30:1037–43. 10.1007/s11064-005-6978-1 16258853

[37] Lafuente H Alvarez FJ Pazos MR Alvarez A Rey-Santano MC Mielgo V. Cannabidiol reduces brain damage and improves functional recovery after acute hypoxia-ischemia in newborn pigs. Pediatr Res. (2011) 70:272. 10.1203/PDR.0b013e3182276b11 21654550

[38] Jones NA Glyn SE Akiyama S Hill TDM Hill AJ Weston SE. Cannabidiol exerts anti-convulsant effects in animal models of temporal lobe and partial seizures. Seizure. (2012) 21:344–52. 10.1016/j.seizure.2012.03.001 22520455

[39] Linge R Jiménez-Sánchez L Campa L Pilar-Cuéllar F Vidal R Pazos A. Cannabidiol induces rapid-acting antidepressant-like effects and enhances cortical 5-HT/glutamate neurotransmission: role of 5-HT1A receptors. Neuropharmacology. (2016) 103:16–26. 10.1016/j.neuropharm.2015.12.017 26711860

[40] Kucerova J Tabiova K Drago F Micale V. Therapeutic potential of cannabinoids in schizophrenia. Recent Pat CNS Drug Discov. (2014) 9:13–25. 10.2174/1574889809666140307115532 24605939

[41] Ruggiero RN Rossignoli MT De Ross JB Hallak JEC Leite JP Bueno-Junior LS. Cannabinoids and vanilloids in schizophrenia: neurophysiological evidence and directions for basic research. Front Pharmacol. (2017) 8:399. 10.3389/fphar.2017.00399 28680405 PMC5478733

[42] Solowij N Broyd SJ Beale C Prick JA Greenwood LM van Hell H. Therapeutic effects of prolonged cannabidiol treatment on psychological symptoms and cognitive function in regular cannabis users: a pragmatic open-label clinical trial. Cannabis Cannabinoid Res. (2018) 3:21–34. 10.1089/can.2017.0043 29607408 PMC5870061

[43] Shannon S Lewis N Lee H Hughes S. Cannabidiol in anxiety and sleep: a large case series. Permanente J. (2019) 23:18–41. 10.7812/TPP/18-041 PMC6326553 30624194

[44] Micale V Drago F. Endocannabinoid system, stress and HPA axis. Eur J Pharmacol. (2018) 834:230–9. 10.1016/j.ejphar.2018.07.039 30036537

[45] Heussler H Cohen J Silove N Tich N Bonn-Miller MO Du W. A phase 1/2, open-label assessment of the safety, tolerability, and efficacy of transdermal cannabidiol (ZYN002) for the treatment of pediatric fragile X syndrome. J Neurodev Disord. (2019) 11:16. 10.1186/s11689-019-9277-x 31370779 PMC6676516

[46] Barchel D Stolar O De-Haan T Ziv-Baran T Saban N Fuchs DO. Oral cannabidiol use in children with autism spectrum disorder to treat related symptoms and co-morbidities. Front Pharmacol. (2019) 9:1521. 10.3389/fphar.2018.01521 30687090 PMC6333745

[47] Devinsky O Cross JH Laux L Marsh E Miller I Nabbout R. Trial of cannabidiol for drug-resistant seizures in the dravet syndrome. N Engl J Med. (2017) 376:2011–20. 10.1056/NEJMoa1611618 28538134

[48] Granata T Marchi N Carlton E Ghosh C Gonzalez-Martinez J Alexopoulos AV. Management of the patient with medically refractory epilepsy. Expert Rev Neurother. (2009) 9:1791–802. 10.1586/ern.09.114 19951138 PMC3761964

[49] Devinsky O Cilio MR Cross H Fernandez-Ruiz J French J Hill C. Cannabidiol: Pharmacology and potential therapeutic role in epilepsy and other neuropsychiatric disorders. Epilepsia. (2014) 55:791–802. 10.1111/epi.12631 24854329 PMC4707667

[50] Jones NA Hill AJ Smith I Bevan SA Williams CM Whalley BJ. Cannabidiol displays antiepileptiform and antiseizure properties in vitro and in vivo. J Pharmacol Exp Ther. (2010) 332:569–77. 10.1124/jpet.109.159145 19906779 PMC2819831

[51] Patra PH Barker-Haliski M White HS Whalley BJ Glyn S Sandhu H. Cannabidiol reduces seizures and associated behavioral comorbidities in a range of animal seizure and epilepsy models. Epilepsia. (2019) 60:303–14. 10.1111/epi.14629 30588604 PMC6378611

[52] Stockings E Zagic D Campbell G Weier M Hall WD Nielsen S. Evidence for cannabis and cannabinoids for epilepsy: a systematic review of controlled and observational evidence. J Neurol Neurosurg Psychiatry. (2018) 89:741–53. 10.1136/jnnp-2017-317168 29511052

[53] Devinsky O Patel AD Cross JH Villanueva V Wirrell EC Privitera M. Effect of cannabidiol on drop seizures in the lennox–gastaut syndrome. N Engl J Med. (2018) 378:1888–97. 10.1056/NEJMoa1714631 29768152

[54] Thiele EA Marsh ED French JA Mazurkiewicz-Beldzinska M Benbadis SR Joshi C. Cannabidiol in patients with seizures associated with Lennox-Gastaut syndrome (GWPCARE4): a randomised, double-blind, placebo-controlled phase 3 trial. Lancet. (2018) 391:1085–96. 10.1016/S0140-6736(18)30136-3 29395273

[55] Perucca E. Cannabinoids in the treatment of epilepsy: hard evidence at last? J Epilepsy Res. (2017) 7:61–76. 10.14581/jer.17012 29344464 PMC5767492

[56] Szaflarski JP Bebin EM Comi AM Patel AD Joshi C Checketts D. Long-term safety and treatment effects of cannabidiol in children and adults with treatment-resistant epilepsies: Expanded access program results. Epilepsia. (2018) 59:1540–8. 10.1111/epi.14477 29998598 PMC6175436

[57] Laprairie RB Bagher AM Kelly MEM Denovan-Wright EM. Cannabidiol is a negative allosteric modulator of the cannabinoid CB1 receptor. Br J Pharmacol. (2015) 172:4790–805. 10.1111/bph.13250 26218440 PMC4621983

[58] Straiker A Dvorakova M Zimmowitch A Mackie K. Cannabidiol inhibits endocannabinoid signaling in autaptic hippocampal neurons. Mol Pharmacol. (2018) 94:743–8. 10.1124/mol.118.111864 29669714 PMC5988021

[59] Burstein SH. Eicosanoid mediation of cannabinoid actions. Bioorg Med Chem. (2019) 27:2718–28. 10.1016/j.bmc.2019.05.018 31104784

[60] Campolongo P Fattore L. Cannabinoid Modulation of Emotion, Memory, and Motivation. New York, NY: Springer (2015). 10.1007/978-1-4939-2294-9

[61] Lu HC Mackie K. An introduction to the endogenous cannabinoid system. Biol Psychiatry. (2016) 79:516–25. 10.1016/j.biopsych.2015.07.028 26698193 PMC4789136

[62] Rodrigues RS Lourenço DM Paulo SL Mateus JM Ferreira MF Mouro FM. Cannabinoid actions on neural stem cells: implications for pathophysiology. Molecules. (2019) 24:1350. 10.3390/molecules24071350 PMC6480122 30959794

[63] Hou L Rong J Haider A Ogasawara D Varlow C Schafroth MA. Positron emission tomography imaging of the endocannabinoid system: opportunities and challenges in radiotracer development. J Med Chem. (2020) 64:123–49. 10.1021/acs.jmedchem.0c01459 33379862 PMC7877880

[64] Busquets-Garcia A Bains J Marsicano G. CB1 receptor signaling in the brain: extracting specificity from ubiquity. Neuropsychopharmacology. (2018) 43:4–20. 10.1038/npp.2017.206 28862250 PMC5719111

[65] Atwood BK Mackie K. CB2: a cannabinoid receptor with an identity crisis. Br J Pharmacol. (2010) 160:467–79. 10.1111/j.1476-5381.2010.00729.x 20590558 PMC2931549

[66] Karhson DS Hardan AY Parker KJ. Endocannabinoid signaling in social functioning: an RDoC perspective. Transl Psychiatry. (2016) 6:e905. 10.1038/tp.2016.169 27676446 PMC5048207

[67] Sawzdargo M Nguyen T Lee DK Lynch KR Cheng R Heng HHQ. Identification and cloning of three novel human G protein-coupled receptor genes GPR52, ΨGPR53 and GPR55: GPR55 is extensively expressed in human brain. Mol Brain Res. (1999) 64:193–8. 10.1016/S0169-328X(98)00277-0 9931487

[68] Ryberg E Larsson N Sjögren S Hjorth S Hermansson NO Leonova J. The orphan receptor GPR55 is a novel cannabinoid receptor. Br J Pharmacol. (2007) 152:1092–101. 10.1038/sj.bjp.0707460 17876302 PMC2095107

[69] Lauckner JE Jensen JB Chen HY Lu HC Hille B Mackie K. GPR55 is a cannabinoid receptor that increases intracellular calcium and inhibits M current. Proc Natl Acad Sci USA. (2008) 105:2699–704. 10.1073/pnas.0711278105 18263732 PMC2268199

[70] Marichal-Cancino BA Fajardo-Valdez A Ruiz-Contreras AE Mendez-Díaz M Prospero-García O. Advances in the physiology of GPR55 in the Central nervous system. Curr Neuropharmacol. (2017) 15:771–8. 10.2174/1570159X14666160729155441 27488130 PMC5771053

[71] Demuth DG Molleman A. Cannabinoid signalling. Life Sci. (2006) 78:549–63. 10.1016/j.lfs.2005.05.055 16109430

[72] Stella N Schweitzer P Piomelli D. A second endogenous cannabinoid that modulates long-term potentiation. Nature. (1997) 388:773–8. 10.1038/42015 9285589

[73] Soltesz I Alger BE Kano M Lee SH Lovinger DM Ohno-Shosaku T. Weeding out bad waves: towards selective cannabinoid circuit control in epilepsy. Nat Rev Neurosci. (2015) 16:264. 10.1038/nrn3937 25891509 PMC10631555

[74] Yu M Ives D Ramesha CS. Synthesis of prostaglandin E2 ethanolamide from anandamide by cyclooxygenase-2. J Biol Chem. (1997) 272:21181–6. 10.1074/jbc.272.34.21181 9261124

[75] Zygmunt PM Petersson J Andersson DA Chuang H Sørgård M Di Marzo V. Vanilloid receptors on sensory nerves mediate the vasodilator action of anandamide. Nature. (1999) 400:452–7. 10.1038/22761 10440374

[76] Storr MA Sharkey KA. The endocannabinoid system and gut–brain signalling. Curr Opin Pharmacol. (2007) 7:575–82. 10.1016/j.coph.2007.08.008 17904903

[77] Di Marzo V. The endocannabinoidome as a substrate for noneuphoric phytocannabinoid action and gut microbiome dysfunction in neuropsychiatric disorders Dialog Clin Neurosci. (2020) 22:259–69. 10.31887/DCNS.2020.22.3/vdimarzo PMC7605024 33162769

[78] Manca C Boubertakh B Leblanc N Deschênes T Lacroix S Martin C. Germ-free mice exhibit profound gut microbiota-dependent alterations of intestinal endocannabinoidome signaling. J Lipid Res. (2020) 61:70–85. 10.1194/jlr.RA119000424 31690638 PMC6939599

[79] Cani PD Plovier H Van Hul M Geurts L Delzenne NM Druart C. Endocannabinoids — at the crossroads between the gut microbiota and host metabolism. Nat Rev Endocrinol. (2016) 12:133–43. 10.1038/nrendo.2015.211 26678807

[80] De Vadder F Kovatcheva-Datchary P Goncalves D Vinera J Zitoun C Duchampt A. Microbiota-generated metabolites promote metabolic benefits via gut-brain neural circuits. Cell. (2014) 156:84–96. 10.1016/j.cell.2013.12.016 24412651

[81] Cohen LJ Esterhazy D Kim SH Lemetre C Aguilar RR Gordon EA. Commensal bacteria make GPCR ligands that mimic human signalling molecules. Nature. (2017) 549:48–53. 10.1038/nature23874 28854168 PMC5777231

[82] Wu C Sun D. GABA receptors in brain development, function, and injury. Metab Brain Dis. (2015) 30:367–79. 10.1007/s11011-014-9560-1 24820774 PMC4231020

[83] Puts NAJ Wodka EL Harris AD Crocetti D Tommerdahl M Mostofsky SH. Reduced GABA and altered somatosensory function in children with autism spectrum disorder. Autism Res. (2017) 10:608–19. 10.1002/aur.1691 27611990 PMC5344784

[84] Sapey-Triomphe LA Lamberton F Sonié S Mattout J Schmitz C. Tactile hypersensitivity and GABA concentration in the sensorimotor cortex of adults with autism. Autism Res. (2019) 12:562–75. 10.1002/aur.2073 30632707

[85] Kolodny T Schallmo MP Gerdts J Edden RAE Bernier RA Murray SO. Concentrations of cortical GABA and glutamate in young adults with autism spectrum disorder. Autism Res. (2020) 13:1111–29. 10.1002/aur.2300 32297709 PMC7387217

[86] Jung KM Sepers M Henstridge CM Lassalle O Neuhofer D Martin H. Uncoupling of the endocannabinoid signalling complex in a mouse model of fragile X syndrome. Nat Commun. (2012) 3:1080. 10.1038/ncomms2045 23011134 PMC3657999

[87] Wei D Dinh D Lee D Li D Anguren A Moreno-Sanz G. Enhancement of anandamide-mediated endocannabinoid signaling corrects autism-related social impairment. Cannabis Cannabinoid Res. (2016) 1:81–9. 10.1089/can.2015.0008 28861483 PMC5549436

[88] Terzian ALB Micale V Wotjak CT. Cannabinoid receptor type 1 receptors on GABAergic vs. glutamatergic neurons differentially gate sex-dependent social interest in mice. Eur J Neurosci. (2014) 40:2293–8. 10.1111/ejn.12561 24698342

[89] Micale V Stepan J Jurik A Pamplona FA Marsch R Drago F. Extinction of avoidance behavior by safety learning depends on endocannabinoid signaling in the hippocampus. J Psychiatr Res. (2017) 90:46–59. 10.1016/j.jpsychires.2017.02.002 28222356

[90] Marsicano G Lutz B. Expression of the cannabinoid receptor CB1 in distinct neuronal subpopulations in the adult mouse forebrain. Eur J Neurosci. (1999) 11:4213–25. 10.1046/j.1460-9568.1999.00847.x 10594647

[91] Ramikie TS Nyilas R Bluett RJ Gamble-George JC Hartley ND Mackie K. Multiple mechanistically distinct modes of endocannabinoid mobilization at central amygdala glutamatergic synapses. Neuron. (2014) 81:1111–25. 10.1016/j.neuron.2014.01.012 24607231 PMC3955008

[92] Ruehle S Remmers F Romo-Parra H Massa F Wickert M Wörtge S. Cannabinoid CB1 receptor in dorsal telencephalic glutamatergic neurons: distinctive sufficiency for hippocampus-dependent and amygdala-dependent synaptic and behavioral functions. J Neurosci. (2013) 33:10264. 10.1523/JNEUROSCI.4171-12.2013 23785142 PMC6618598

[93] Häring M Marsicano G Lutz B Monory K. Identification of the cannabinoid receptor type 1 in serotonergic cells of raphe nuclei in mice. Neuroscience. (2007) 146:1212–9. 10.1016/j.neuroscience.2007.02.021 17383106

[94] Llorente-Berzal A Terzian AL di Marzo V Micale V Viveros MP Wotjak CT. 2-AG promotes the expression of conditioned fear via cannabinoid receptor type 1 on GABAergic neurons. Psychopharmacology. (2015) 232:2811–25. 10.1007/s00213-015-3917-y 25814137

[95] Terzian AL Drago F Wotjak C Micale V. The dopamine and cannabinoid interaction in the modulation of emotions and cognition: assessing the role of cannabinoid CB1 receptor in neurons expressing dopamine D1 receptors. Front Behav Neurosci. (2011) 5:49. 10.3389/fnbeh.2011.00049 21887137 PMC3156975

[96] Gerdeman G Lovinger DM. CB1 cannabinoid receptor inhibits synaptic release of glutamate in rat dorsolateral striatum. J Neurophysiol. (2001) 85:468–71. 10.1152/jn.2001.85.1.468 11152748

[97] Katona I Sperlágh B Sík A Käfalvi A Vizi ES Mackie K. Presynaptically located CB1 cannabinoid receptors regulate GABA release from axon terminals of specific hippocampal interneurons. J Neurosci. (1999) 19:4544–58. 10.1523/JNEUROSCI.19-11-04544.1999 10341254 PMC6782612

[98] Ativie F Komorowska JA Beins E Albayram Ö Zimmer T Zimmer A. Cannabinoid 1 receptor signaling on hippocampal GABAergic neurons influences microglial activity. Front Mol Neurosci. (2018) 11:295. 10.3389/fnmol.2018.00295 30210289 PMC6121063

[99] Liu X Dimidschstein J Fishell G Carter AG. Hippocampal inputs engage CCK+ interneurons to mediate endocannabinoid-modulated feed-forward inhibition in the prefrontal cortex. eLife. (2020) 9:e55267. 10.7554/eLife.55267.sa2 33034285 PMC7609047

[100] Patel S Hill MN Cheer JF Wotjak CT Holmes A. The endocannabinoid system as a target for novel anxiolytic drugs. Neurosci Biobehav Rev. (2017) 76:56–66. 10.1016/j.neubiorev.2016.12.033 28434588 PMC5407316

[101] Gobbi G Bambico FR Mangieri R Bortolato M Campolongo P Solinas M. Antidepressant-like activity and modulation of brain monoaminergic transmission by blockade of anandamide hydrolysis. Proc Natl Acad Sci USA. (2005) 102:18620–5. 10.1073/pnas.0509591102 16352709 PMC1317988

[102] Busquets-Garcia A Puighermanal E Pastor A de la Torre R Maldonado R Ozaita A. Differential role of anandamide and 2-arachidonoylglycerol in memory and anxiety-like responses. Biol Psychiatry. (2011) 70:479–86. 10.1016/j.biopsych.2011.04.022 21684528

[103] Ivy D Palese F Vozella V Fotio Y Yalcin A Ramirez G. Cannabinoid CB2 receptors mediate the anxiolytic-like effects of monoacylglycerol lipase inhibition in a rat model of predator-induced fear. Neuropsychopharmacology. (2020) 45:1330–8. 10.1038/s41386-020-0696-x 32375160 PMC7298057

[104] Vezzani A Balosso S Ravizza T. Neuroinflammatory pathways as treatment targets and biomarkers in epilepsy. Nat Rev Neurol. (2019) 15:459–72. 10.1038/s41582-019-0217-x 31263255

[105] Lee BH Smith T Paciorkowski AR. Autism spectrum disorder and epilepsy: disorders with a shared biology. Epilepsy Behav. (2015) 47:191–201. 10.1016/j.yebeh.2015.03.017 25900226 PMC4475437

[106] Wither RG Borlot F MacDonald A Butcher NJ Chow EWC Bassett AS. 22q11.2 deletion syndrome lowers seizure threshold in adult patients without epilepsy. Epilepsia. (2017) 58:1095–101. 10.1111/epi.13748 28448680

[107] Mudigoudar B Nune S Fulton S Dayyat E Wheless JW. Epilepsy in 22q11.2 deletion syndrome: a case series and literature review. Pediatr Neurol. (2017) 76:86–90. 10.1016/j.pediatrneurol.2017.08.011 28969878

[108] Dale T Downs J Olson H Bergin AM Smith S Leonard H. Cannabis for refractory epilepsy in children: a review focusing on CDKL5 deficiency disorder. Epilepsy Res. (2019) 151:31–9. 10.1016/j.eplepsyres.2019.02.001 30771550

[109] Rojas A Chen D Ganesh T Varvel NH Dingledine R. The COX-2/prostanoid signaling cascades in seizure disorders. Expert Opin Ther Targets. (2019) 23:1–13. 10.1080/14728222.2019.1554056 30484341 PMC6481174

[110] Vargas DL Nascimbene C Krishnan C Zimmerman AW Pardo CA. Neuroglial activation and neuroinflammation in the brain of patients with autism. Ann Neurol. (2005) 57:67–81. 10.1002/ana.20315 15546155

[111] Jyonouchi H Sun S Le H. Proinflammatory and regulatory cytokine production associated with innate and adaptive immune responses in children with autism spectrum disorders and developmental regression. J Neuroimmunol. (2001) 120:170–9. 10.1016/S0165-5728(01)00421-0 11694332

[112] Hassan S Eldeeb K Millns PJ Bennett AJ Alexander SPH Kendall DA. Cannabidiol enhances microglial phagocytosis via transient receptor potential (TRP) channel activation. Br J Pharmacol. (2014) 171:2426–39. 10.1111/bph.12615 24641282 PMC3997281

[113] Ravizza T Boer K Redeker S Spliet WGM van Rijen PC Troost D. The IL-1β system in epilepsy-associated malformations of cortical development. Neurobiol Dis. (2006) 24:128–43. 10.1016/j.nbd.2006.06.003 16860990

[114] Vezzani A Viviani B. Neuromodulatory properties of inflammatory cytokines and their impact on neuronal excitability. Neuropharmacology. (2015) 96:70–82. 10.1016/j.neuropharm.2014.10.027 25445483

[115] Vezzani A Lang B Aronica E. Immunity and inflammation in epilepsy. Cold Spring Harbor Perspect Med. (2015) 6:a022699. 10.1101/cshperspect.a022699 PMC4743070 26684336

[116] Pietr M Kozela E Levy R Rimmerman N Lin YH Stella N. Differential changes in GPR55 during microglial cell activation. FEBS Lett. (2009) 583:2071–6. 10.1016/j.febslet.2009.05.028 19464294

[117] Araujo DJ Tjoa K Saijo K. The endocannabinoid system as a window into microglial biology and its relationship to autism. Front Cell Neurosci. (2019) 13:424. 10.3389/fncel.2019.00424 31619967 PMC6759510

[118] Morgan JT Chana G Pardo CA Achim C Semendeferi K Buckwalter J. Microglial activation and increased microglial density observed in the dorsolateral prefrontal cortex in autism. Biol Psychiatry. (2010) 68:368–76. 10.1016/j.biopsych.2010.05.024 20674603

[119] Suzuki K Sugihara G Ouchi Y Nakamura K Futatsubashi M Takebayashi K. Microglial activation in young adults with autism spectrum disorder. JAMA Psychiatry. (2013) 70:49–58. 10.1001/jamapsychiatry.2013.272 23404112

[120] Fox A Bevan S. Therapeutic potential of cannabinoid receptor agonists as analgesic agents. Expert Opin Investig Drugs. (2005) 14:695–703. 10.1517/13543784.14.6.695 16004597

[121] Gleave RJ Beswick PJ Brown AJ Giblin GMP Goldsmith P Haslam CP. Synthesis and evaluation of 3-amino-6-aryl-pyridazines as selective CB2 agonists for the treatment of inflammatory pain. Bioorg Med Chem Lett. (2010) 20:465–8. 10.1016/j.bmcl.2009.11.117 20005703

[122] Cantarella G Scollo M Lempereur L Saccani-Jotti G Basile F Bernardini R. Endocannabinoids inhibit release of nerve growth factor by inflammation-activated mast cells. Biochem Pharmacol. (2011) 82:380–8. 10.1016/j.bcp.2011.05.004 21601562

[123] Borrelli F Romano B Petrosino S Pagano E Capasso R Coppola D. Palmitoylethanolamide, a naturally occurring lipid, is an orally effective intestinal anti-inflammatory agent. Br J Pharmacol. (2015) 172:142–58. 10.1111/bph.12907 25205418 PMC4280974

[124] Staton PC Hatcher JP Walker DJ Morrison AD Shapland EM Hughes JP. The putative cannabinoid receptor GPR55 plays a role in mechanical hyperalgesia associated with inflammatory and neuropathic pain. Pain. (2008) 139:225–36. 10.1016/j.pain.2008.04.006 18502582

[125] Saliba SW Jauch H Gargouri B Keil A Hurrle T Volz N. Anti-neuroinflammatory effects of GPR55 antagonists in LPS-activated primary microglial cells. J Neuroinflamm. (2018) 15:322–2. 10.1186/s12974-018-1362-7 PMC6240959 30453998

[126] Hill JD Zuluaga-Ramirez V Gajghate S Winfield M Sriram U Rom S. Activation of GPR55 induces neuroprotection of hippocampal neurogenesis and immune responses of neural stem cells following chronic, systemic inflammation. Brain Behav Immun. (2019) 76:165–81. 10.1016/j.bbi.2018.11.017 30465881 PMC6398994

[127] Kallendrusch S Kremzow S Nowicki M Grabiec U Winkelmann R Benz A. The G protein-coupled receptor 55 ligand l-α-lysophosphatidylinositol exerts microglia-dependent neuroprotection after excitotoxic lesion. Glia. (2013) 61:1822–31. 10.1002/glia.22560 24038453

[128] Alhouayek M Muccioli GG. COX-2-derived endocannabinoid metabolites as novel inflammatory mediators. Trends Pharmacol Sci. (2014) 35:284–92. 10.1016/j.tips.2014.03.001 24684963

[129] Kozak KR Rowlinson SW Marnett LJ. Oxygenation of the endocannabinoid, 2-arachidonylglycerol, to glyceryl prostaglandins by cyclooxygenase-2. J Biol Chem. (2000) 275:33744–9. 10.1074/jbc.M007088200 10931854

[130] Carrasco E Casper D Werner P. PGE2 receptor EP1 renders dopaminergic neurons selectively vulnerable to low-level oxidative stress and direct PGE2 neurotoxicity. J Neurosci Res. (2007) 85:3109–17. 10.1002/jnr.21425 17868147

[131] Saleem S Ahmad AS Maruyama T Narumiya S Doré S. PGF2α FP receptor contributes to brain damage following transient focal brain ischemia. Neurotox Res. (2009) 15:62–70. 10.1007/s12640-009-9007-3 19384589 PMC6010178

[132] Nomura DK Morrison BE Blankman JL Long JZ Kinsey SG Marcondes MCG. Endocannabinoid hydrolysis generates brain prostaglandins that promote neuroinflammation. Science. (2011) 334:809–13. 10.1126/science.1209200 22021672 PMC3249428

[133] Desjardins P Sauvageau A Bouthillier A Navarro D Hazell AS Rose C. Induction of astrocytic cyclooxygenase-2 in epileptic patients with hippocampal sclerosis. Neurochem Int. (2003) 42:299–303. 10.1016/S0197-0186(02)00101-8 12470703

[134] Serrano GE Lelutiu N Rojas A Cochi S Shaw R Makinson CD. Ablation of cyclooxygenase-2 in forebrain neurons is neuroprotective and dampens brain inflammation after status epilepticus. J Neurosci. (2011) 31:14850–60. 10.1523/JNEUROSCI.3922-11.2011 22016518 PMC4126152

[135] Ravindranath V Strobel HW. Cytochrome P450-mediated metabolism in brain: functional roles and their implications. Expert Opin Drug Metab Toxicolo. (2013) 9:551–8. 10.1517/17425255.2013.759208 23330950

[136] Agarwal V Kommaddi RP Valli K Ryder D Hyde TM Kleinman JE. Drug metabolism in human brain: high levels of cytochrome P4503A43 in brain and metabolism of anti-anxiety drug alprazolam to its active metabolite. PLoS ONE. (2008) 3:e2337. 10.1371/journal.pone.0002337 18545703 PMC2408964

[137] Pai HV Kommaddi RP Chinta SJ Mori T Boyd MR Ravindranath V. A frameshift mutation and alternate splicing in human brain generate a functional form of the pseudogene cytochrome P4502D7 That demethylates codeine to morphine. J Biol Chem. (2004) 279:27383–9. 10.1074/jbc.M402337200 15051713

[138] Li N Liu JY Timofeyev V Qiu H Hwang SH Tuteja D. Beneficial effects of soluble epoxide hydrolase inhibitors in myocardial infarction model: Insight gained using metabolomic approaches. J Mol Cell Cardiol. (2009) 47:835–45. 10.1016/j.yjmcc.2009.08.017 19716829 PMC3290524

[139] Node K Huo Y Ruan X Yang B Spiecker M Ley K. Anti-inflammatory properties of cytochrome P450 epoxygenase-derived eicosanoids. Science. (1999) 285:12769. 10.1126/science.285.5431.1276 PMC2720027 10455056

[140] Westphal C Konkel A Schunck WH. CYP-eicosanoids—A new link between omega-3 fatty acids and cardiac disease? Prostaglandins Other Lipid Mediat. (2011) 96:99–108. 10.1016/j.prostaglandins.2011.09.001 21945326

[141] Romashko M Schragenheim J Abraham NG McClung JA. Epoxyeicosatrienoic acid as therapy for diabetic and ischemic cardiomyopathy. Trends Pharmacol Sci. (2016) 37:945–62. 10.1016/j.tips.2016.08.001 27633970

[142] Snider NT Kornilov AM Kent UM Hollenberg PF. Anandamide metabolism by human liver and kidney microsomal cytochrome P450 enzymes to form hydroxyeicosatetraenoic and epoxyeicosatrienoic acid ethanolamides. J Pharmacol Experi Therap. (2007) 321:590. 10.1124/jpet.107.119321 17272674

[143] Snider NT Sridar C Hollenberg P. The endocannabinoid anandamide is a substrate of cytochrome P450 2D6. FASEB J. (2008) 22:920. 10.1124/jpet.108.141796 PMC2704579 18698000

[144] Walker VJ Griffin AP Hammar DK Hollenberg PF. Metabolism of anandamide by human cytochrome P450 2J2 in the reconstituted system and human intestinal microsomes. J Pharmacol Exp Ther. (2016) 357, 537–544. 10.1124/jpet.116.232553 27000802 PMC4885506

[145] Snider NT Nast JA Tesmer LA Hollenberg PF. A cytochrome P450-derived epoxygenated metabolite of anandamide is a potent cannabinoid receptor 2-selective agonist. Mol Pharmacol. (2009) 75:965. 10.1124/mol.108.053439 19171674 PMC2684935

[146] Sridar C Snider NT Hollenberg PF. Anandamide oxidation by wild-type and polymorphically expressed CYP2B6 and CYP2D6. Drug Metab Disposit. (2011) 39:782. 10.1124/dmd.110.036707 PMC3082373 21289075

[147] McDougle DR Kambalyal A Meling DD Das A. Endocannabinoids anandamide and 2-arachidonoylglycerol are substrates for human CYP2J2 epoxygenase. J Pharmacol Experi Therap. (2014) 351:616. 10.1124/jpet.114.216598 PMC4244587 25277139

[148] Chen JK Chen J Imig JD Wei S Hachey DL Guthi JS. Identification of novel endogenous cytochrome P450 arachidonate metabolites with high affinity for cannabinoid receptors. J Biol Chem. (2008) 283:24514–24. 10.1074/jbc.M709873200 18606824 PMC2528993

[149] Ghosh A Chen F Thakur A Hong H. Cysteinyl leukotrienes and their receptors: emerging therapeutic targets in central nervous system disorders. CNS Neurosci Therap. (2016) 22:943–51. 10.1111/cns.12596 27542570 PMC6492851

[150] Drazen JM Israel E O'Byrne PM. Treatment of asthma with drugs modifying the leukotriene pathway. N Engl J Med. (1999) 340:197–206. 10.1056/NEJM199901213400306 9895400

[151] Lenz QF Arroyo DS Temp FR Poersch AB Masson CJ Jesse AC. Cysteinyl leukotriene receptor (CysLT) antagonists decrease pentylenetetrazol-induced seizures and blood–brain barrier dysfunction. Neuroscience. (2014) 277:859–71. 10.1016/j.neuroscience.2014.07.058 25090924

[152] Gorter JA Aronica E van Vliet EA. The roof is leaking and a storm is raging: repairing the blood–brain barrier in the fight against epilepsy. Epilepsy Currents. (2019) 19:177–81. 10.1177/1535759719844750 31037960 PMC6610387

[153] Yu S Zhang X Wang X Xu D Chen L Zhang L. Cysteinyl leukotriene receptor 1 mediates LTD4-induced activation of mouse microglial cells *in vitro*. Acta Pharmacol Sin. (2014) 35:33–40. 10.1038/aps.2013.130 24141567 PMC4075749

[154] McElhanon BO McCracken C Karpen S Sharp WG. Gastrointestinal symptoms in autism spectrum disorder: a meta-analysis. Pediatrics. (2014) 133:872–83. 10.1542/peds.2013-3995 24777214

[155] Hsiao EY. Gastrointestinal issues in autism spectrum disorder. Harv Rev Psychiatry. (2014) 22:104–11. 10.1097/HRP.0000000000000029 24614765

[156] Furuta GT Williams K Kooros K Kaul A Panzer R Coury DL. Management of constipation in children and adolescents with autism spectrum disorders. Pediatrics. (2012) 130(Suppl. 2):S98–105. 10.1542/peds.2012-0900H 23118260

[157] Gorrindo P Williams KC Lee EB Walker LS McGrew SG Levitt P. Gastrointestinal dysfunction in autism: parental report, clinical evaluation, associated factors. Autism Res. (2012) 5:101–8. 10.1002/aur.237 22511450 PMC3335766

[158] Strati F Cavalieri D Albanese D De Felice C Donati C Hayek J. New evidences on the altered gut microbiota in autism spectrum disorders. Microbiome. (2017) 5:24. 10.1186/s40168-017-0242-1 28222761 PMC5320696

[159] Minter MR Zhang C Leone V Ringus DL Zhang X Oyler-Castrillo P. Antibiotic-induced perturbations in gut microbial diversity influences neuro-inflammation and amyloidosis in a murine model of Alzheimer's disease. Sci Rep. (2016) 6:30028. 10.1038/srep30028 27443609 PMC4956742

[160] Parker A Fonseca S Carding SR. Gut microbes and metabolites as modulators of blood-brain barrier integrity and brain health. Gut Microbes. (2020) 11:135–57. 10.1080/19490976.2019.1638722 31368397 PMC7053956

[161] Tang W Zhu H Feng Y Guo R Wan D. The impact of gut microbiota disorders on the blood-brain barrier. Infect Drug Resistance. (2020) 13:3351–63. 10.2147/IDR.S254403 PMC7532923 33061482

[162] Tham M Yilmaz O Alaverdashvili M Kelly MEM Denovan-Wright EM Laprairie RB. Allosteric and orthosteric pharmacology of cannabidiol and cannabidiol-dimethylheptyl at the type 1 and type 2 cannabinoid receptors. Br J Pharmacol. (2019) 176:1455–69. 10.1111/bph.14440 29981240 PMC6487556

[163] Thomas A Baillie GL Phillips AM Razdan RK Ross RA Pertwee RG. Cannabidiol displays unexpectedly high potency as an antagonist of CB1 and CB2 receptor agonists *in vitro*. Br J Pharmacol. (2007) 150:613–23. 10.1038/sj.bjp.0707133 17245363 PMC2189767

[164] Kaplan JS Stella N Catterall WA Westenbroek RE. Cannabidiol attenuates seizures and social deficits in a mouse model of dravet syndrome. Proc Natl Acad Sci USA. (2017) 114:11229–34. 10.1073/pnas.1711351114 28973916 PMC5651774

[165] Friedman D Devinsky O. Cannabinoids in the treatment of epilepsy. N Engl J Med. (2015) 373:1048–58. 10.1056/NEJMra1407304 26352816

[166] Katona I. Cannabis and endocannabinoid signaling in epilepsy. In: Pertwee RG, editor. Endocannabinoids. Cham: Springer International Publishing (2015). p. 285–316. 10.1007/978-3-319-20825-1_10 26408165

[167] Bouron A. Phyto and endocannabinoids exert complex actions on calcium and zinc signaling in mouse cortical neurons. Biochem Pharmacol. (2018) 152:244–51. 10.1016/j.bcp.2018.04.003 29630867

[168] Romigi A Bari M Placidi F Marciani MG Malaponti M Torelli F. Cerebrospinal fluid levels of the endocannabinoid anandamide are reduced in patients with untreated newly diagnosed temporal lobe epilepsy. Epilepsia. (2010) 51:768–72. 10.1111/j.1528-1167.2009.02334.x 19817812

[169] Aran A Eylon M Harel M Polianski L Nemirovski A Tepper S. Lower circulating endocannabinoid levels in children with autism spectrum disorder. Mol Autism. (2019) 10:11. 10.1186/s13229-019-0256-6 30728928 PMC6354384

[170] Karhson DS Krasinska KM Dallaire JA Libove RA Phillips JM Chien AS. Plasma anandamide concentrations are lower in children with autism spectrum disorder. Mol Autism. (2018) 9:18. 10.1186/s13229-018-0203-y 29564080 PMC5848550

[171] Elmes MW Kaczocha M Berger WT Leung K Ralph BP Wang L. Fatty Acid Binding Proteins (FABPs) are Intracellular Carriers for Δ9-Tetrahydrocannabinol (THC) and Cannabidiol (CBD). J Biol Chem. (2015) 290:8711–21. 10.1074/jbc.M114.618447 25666611 PMC4423662

[172] De Petrocellis L Ligresti A Moriello AS Allarà M Bisogno T Petrosino S. Effects of cannabinoids and cannabinoid-enriched Cannabis extracts on TRP channels and endocannabinoid metabolic enzymes. Br J Pharmacol. (2011) 163:1479–94. 10.1111/j.1476-5381.2010.01166.x 21175579 PMC3165957

[173] Wei D Lee D Cox CD Karsten CA Peñagarikano O Geschwind DH. Endocannabinoid signaling mediates oxytocin-driven social reward. Proc Natl Acad Sci USA. (2015) 112:14084–9. 10.1073/pnas.1509795112 26504214 PMC4653148

[174] DeVuono MV Hrelja KM Sabaziotis L Rajna A Rock EM Limebeer CL. Conditioned gaping produced by high dose Δ9-tetrahydracannabinol: Dysregulation of the hypothalamic endocannabinoid system. Neuropharmacology. (2018) 141:272–82. 10.1016/j.neuropharm.2018.08.039 30195587

[175] Chang YH Lee ST Lin WW. Effects of cannabinoids on LPS-stimulated inflammatory mediator release from macrophages: Involvement of eicosanoids. J Cell Biochem. (2001) 81:715–23. 10.1002/jcb.1103 11329626

[176] Ruhaak LR Felth J Karlsson PC Rafter JJ Verpoorte R Bohlin L. Evaluation of the cyclooxygenase inhibiting effects of six major cannabinoids isolated from cannabis sativa. Biol Pharm Bull. (2011) 34:774–8. 10.1248/bpb.34.774 21532172

[177] Bornheim LM Kim KY Chen BL Correia MA. The effect of cannabidiol on mouse hepatic microsomal cytochrome P450-dependent anandamide metabolism. Biochem Biophys Res Commun. (1993) 197:740–6. 10.1006/bbrc.1993.2541 8267610

[178] Arnold WR Weigle AT Das A. Cross-talk of cannabinoid and endocannabinoid metabolism is mediated via human cardiac CYP2J2. J Inorg Biochem. (2018) 184:88–99. 10.1016/j.jinorgbio.2018.03.016 29689453 PMC5964033

[179] Massi P Valenti M Vaccani A Gasperi V Perletti G Marras E. 5-Lipoxygenase and anandamide hydrolase (FAAH) mediate the antitumor activity of cannabidiol, a non-psychoactive cannabinoid. J Neurochem. (2008) 104:1091–100. 10.1111/j.1471-4159.2007.05073.x 18028339

[180] Fleck J Temp FR Marafiga JR Jesse AC Milanesi LH Rambo LM. Montelukast reduces seizures in pentylenetetrazol-kindled mice. Brazil J Med Biol Res. (2016) 49:e5031. 10.1590/1414-431X20155031 PMC4792507 26909785

[181] Rehni AK Singh TG. Modulation of leukotriene D4 attenuates the development of seizures in mice. Prostaglandins Leukotrienes Essential Fatty Acids. (2011) 85:97–106. 10.1016/j.plefa.2011.04.003 21641195

[182] Takahashi Y Imai K Ikeda H Kubota Y Yamazaki E Susa F. Open study of pranlukast add-on therapy in intractable partial epilepsy. Brain Dev. (2013) 35:236–44. 10.1016/j.braindev.2012.04.001 22571867

[183] Iannotti FA Hill CL Leo A Alhusaini A Soubrane C Mazzarella E. Nonpsychotropic plant cannabinoids, cannabidivarin (CBDV) and cannabidiol (CBD), activate and desensitize transient receptor potential vanilloid 1 (TRPV1) channels *in vitro*: potential for the treatment of neuronal hyperexcitability. ACS Chem Neurosci. (2014) 5:1131–41. 10.1021/cn5000524 25029033

[184] Limebeer CL Rock EM Sharkey KA Parker LA. Nausea-induced 5-HT release in the interoceptive insular cortex and regulation by monoacylglycerol lipase (MAGL) inhibition and cannabidiol. eNeuro. (2018) 5:ENEURO.0256-0218.2018. 10.1523/ENEURO.0256-18.2018 PMC6071201 30073198

[185] Ibarra-Lecue I Mollinedo-Gajate I Meana JJ Callado LF Diez-Alarcia R Urigüen L. Chronic cannabis promotes pro-hallucinogenic signaling of 5-HT2A receptors through Akt/mTOR pathway. Neuropsychopharmacology. (2018) 43:2028–35. 10.1038/s41386-018-0076-y 29748632 PMC6098160

[186] Pretzsch CM Freyberg J Voinescu B Lythgoe D Horder J Mendez MA. Effects of cannabidiol on brain excitation and inhibition systems; a randomised placebo-controlled single dose trial during magnetic resonance spectroscopy in adults with and without autism spectrum disorder. Neuropsychopharmacology. (2019) 44:1398–405. 10.1038/s41386-019-0333-8 30758329 PMC6784992

[187] Stott CG Nichol K Jones NA Gray RA Bazelot M Whalley BJ. The proposed multimodal mechanism of action of cannabidiol (CBD) in epilepsy: Modulation of intracellular calcium and adenosine-mediated signalling. Epilepsy Behav. (2019) 101:106734. 10.1016/j.yebeh.2019.08.009

[188] Pandolfo P Silveirinha V Santos-Rodrigues A Venance L Ledent C Takahashi RN. Cannabinoids inhibit the synaptic uptake of adenosine and dopamine in the rat and mouse striatum. Eur J Pharmacol. (2011) 655:38–45. 10.1016/j.ejphar.2011.01.013 21266173

[189] Carrier EJ Auchampach JA Hillard CJ. Inhibition of an equilibrative nucleoside transporter by cannabidiol: a mechanism of cannabinoid immunosuppression. Proc Natl Acad Sci USA. (2006) 103:7895–900. 10.1073/pnas.0511232103 16672367 PMC1472541

[190] Pellati F Borgonetti V Brighenti V Biagi M Benvenuti S Corsi L. Cannabis sativa L. and nonpsychoactive cannabinoids: their chemistry and role against oxidative stress, inflammation, and cancer. BioMed Res Int. (2018) 2018:1691428. 10.1155/2018/1691428 30627539 PMC6304621

[191] Atalay S Jarocka-Karpowicz I Skrzydlewska E. Antioxidative and anti-inflammatory properties of cannabidiol. Antioxidants. (2020) 9:21. 10.3390/antiox9010021 PMC7023045 31881765

[192] Rimmerman N Ben-Hail D Porat Z Juknat A Kozela E Daniels MP. Direct modulation of the outer mitochondrial membrane channel, voltage-dependent anion channel 1 (VDAC1) by cannabidiol: a novel mechanism for cannabinoid-induced cell death. Cell Death Dis. (2013) 4:e949. 10.1038/cddis.2013.471 24309936 PMC3877544

[193] Bakas T van Nieuwenhuijzen PS Devenish SO McGregor IS Arnold JC Chebib M. The direct actions of cannabidiol and 2-arachidonoyl glycerol at GABAA receptors. Pharmacol Res. (2017) 119:358–70. 10.1016/j.phrs.2017.02.022 28249817

[194] Anderson LL Absalom NL Abelev SV Low IK Doohan PT Martin LJ. Coadministered cannabidiol and clobazam: preclinical evidence for both pharmacodynamic and pharmacokinetic interactions. Epilepsia. (2019) 60:2224–34. 10.1111/epi.16355 31625159 PMC6900043

[195] Lozano R Martinez-Cerdeno V Hagerman RJ. Advances in the understanding of the gabaergic neurobiology of FMR1 expanded alleles leading to targeted treatments for fragile X spectrum disorder. Curr Pharm Des. (2015) 21:4972–79. 10.2174/1381612821666150914121038 26365141 PMC4830341

[196] D'Addario C Micale V Di Bartolomeo M Stark T Pucci M Sulcova A. A preliminary study of endocannabinoid system regulation in psychosis: distinct alterations of CNR1 promoter DNA methylation in patients with schizophrenia. Schizophr Res. (2017) 188:132–40. 10.1016/j.schres.2017.01.022 28108228

[197] Di Bartolomeo M Stark T Maurel OM Iannotti FA Kuchar M Ruda-Kucerova J. Crosstalk between the transcriptional regulation of dopamine D2 and cannabinoid CB1 receptors in schizophrenia: Analyses in patients and in perinatal Δ9-tetrahydrocannabinol-exposed rats. Pharmacol Res. (2021) 164:105357. 10.1016/j.phrs.2020.105357 33285233

[198] Stark T Di Bartolomeo M Di Marco R Drazanova E Platania CBM Iannotti FA. Altered dopamine D3 receptor gene expression in MAM model of schizophrenia is reversed by peripubertal cannabidiol treatment. Biochem Pharmacol. (2020) 177:114004. 10.1016/j.bcp.2020.114004 32360362

[199] Stark T Ruda-Kucerova J Iannotti FA D'Addario C Di Marco R Pekarik V. Peripubertal cannabidiol treatment rescues behavioral and neurochemical abnormalities in the MAM model of schizophrenia. Neuropharmacology. (2019) 146:212–221. 10.1016/j.neuropharm.2018.11.035 30496751

[200] Taylor L Gidal B Blakey G Tayo B Morrison G. A phase I. Randomized, double-blind, placebo-controlled, single ascending dose, multiple dose, and food effect trial of the safety, tolerability and pharmacokinetics of highly purified cannabidiol in healthy subjects. CNS Drugs. (2018) 32:1053–67. 10.1007/s40263-018-0578-5 30374683 PMC6223703

[201] Iannotti FA Di Marzo V. The gut microbiome, endocannabinoids and metabolic disorders. J Endocrinol. (2021) 248:R83–97. 10.1530/JOE-20-0444 33337346

[202] Pagano E Capasso R Piscitelli F Romano B Parisi OA Finizio S. An orally active cannabis extract with high content in cannabidiol attenuates chemically-induced intestinal inflammation and hypermotility in the mouse. Front Pharmacol. (2016) 7:341. 10.3389/fphar.2016.00341 27757083 PMC5047908

[203] Al-Ghezi ZZ Busbee PB Alghetaa H Nagarkatti PS Nagarkatti M. Combination of cannabinoids, delta-9-tetrahydrocannabinol (THC) and cannabidiol (CBD), mitigates experimental autoimmune encephalomyelitis (EAE) by altering the gut microbiome. Brain Behav Immun. (2019) 82:25–35. 10.1016/j.bbi.2019.07.028 31356922 PMC6866665

